# Seasonal variation in the lipid content of Fraser River Chinook Salmon (*Oncorhynchus tshawytscha)* and its implications for Southern Resident Killer Whale (*Orcinus orca*) prey quality

**DOI:** 10.1038/s41598-023-28321-9

**Published:** 2023-02-15

**Authors:** Jacob E. Lerner, Brian P. V. Hunt

**Affiliations:** 1 Institute for the Oceans and Fisheries, University of British Columbia, Vancouver, BC Canada; 2 Department of Earth, Ocean and Atmospheric Sciences, University of British Columbia, Vancouver, BC Canada; 3 Hakai Institute, Heriot Bay, BC Canada

**Keywords:** Marine biology, Ecology, Ecology, Biooceanography, Conservation biology

## Abstract

In Southern British Columbia (BC), Canada, declines in southern resident killer whale (SRKW—*Orcinus orca*) populations have been linked to declines in numbers and average size of their preferred prey, Chinook salmon (*Oncorhynchus tshawytscha*). However, the life history diversity of Chinook suggests that there is a need to assess stock-specific differences in energy density to evaluate prey quality as a factor in SRKW declines. In this study, we calibrated a Distell fat meter to estimate Chinook whole-body lipid content, a proxy for energy density. The fat meter was deployed at the Fraser River, BC, Chinook test fishery during 2020, collecting lipid, weight, and length measurements from 1566 genetically stock identified individuals encompassing all major Fraser River Chinook population units (management units, MUs) at river entry. We found that MU-specific lipid content increased with distance and elevation to spawning grounds and was highest in the Spring-5_2_ (12.8%) and Summer-5_2_ (12.7%) MUs, intermediate in the Summer-4_1_ MU (10.8%), and lowest in the Fall-4_1_ MU (7.3%). Lipid content also decreased by up to 6 percentage points within MUs from the beginning to end of their migration period. Our data revealed SRKWs’ most endangered prey sources, the Spring-5_2_ and Summer-5_2_ MUs, are also its most energy rich. It also indicated SRKWs have access to progressively lower energy density Chinook through the year, requiring up to ~ 30% more fish to meet energy demands in the fall than in the spring.

## Introduction

A critical step in the recovery of an endangered species is identifying the cause of the species’ decline^1^. Once isolated, the agent of decline can be addressed to arrest the collapse of the species. Significant effort has been expended to identify the cause of the decline/stagnation of the endangered southern resident killer whales (SRKWs), *Orcinus orca*, a population of northeastern Pacific killer whales found from the Salish Sea south to California^2,3^. Though its small population size and low genetic variation inherently puts the SRKW population at risk^4^, this is not thought to have induced their recent population struggles^3^. Instead, other potential suspects in their recent decline have been considered. These include the effects of anthrophonic noise^5^ and contaminant accumulation^6^, yet much of the blame has fallen on a concurrent decline in the abundance of Chinook salmon, *Oncorhynchus tshawytscha*, their preferred prey^7,8^.

Resident killer whales, including both the SRKWs and their northern relatives (NRKWs), are an ecotype of northeastern Pacific killer whales that specialize in fish predation, specifically Chinook salmon^8–10^. Chinook are favoured prey in part because they are large and coastally distributed, but predominantly because they have a very high energy density^11,12^. Their energy density can be 50% greater than that of chum salmon (*Oncorhynchus keta*), an only occasional prey of SRKWs. Chinook salmon’s high energy density is driven by the high levels of lipid they accumulate at sea, a necessity to sustain their freshwater spawning migrations and sexual maturation^11^.

Across the west coast, both Chinook absolute numbers and average size are in decline^13–16^. This has created a compelling narrative: that declines in both Chinook size and number have caused population declines amongst SRKWs^10^. However, in the summer up to 90% of SRKW diet is comprised of Chinook from the Fraser River with Puget Sound origin Chinook forming a large part of their diet in the fall^17^, and among many of these Chinook populations there are discrepancies to the abovementioned negative trends that do not comport with this thesis. For example, when hatchery populations are included, Chinook abundances in Juan de Fuca Strait and Puget Sound have increased or remained steady over the past four decades, albeit body mass has decreased^18^. Beyond Puget Sound, Chinook caught in British Columbia (BC) buck a coastwide trend in declines in size-at-age and do not show recent evidence of getting smaller^13,15^. On the Fraser River, middle and upper river Chinook populations have experienced substantial declines in recent years^19^, yet two of the historically largest Chinook populations on the Fraser, the Summer South Thompson and Fall Harrison River populations, remained healthy as recently as 2016^20^. Of these two populations, the Summer South Thompson is near all-time highs and, although the Fall Harrison River population has experienced a decline in the past 6 years, this drop occurred *after* SRKW populations began to decline in the mid-1990s^21,22^.

What these discrepancies suggest is that we do not yet have complete information on the cause of the SRKW decline/stagnation. One area in particular that requires more research is the prey quality of the Chinook themselves, defined by their lipid content and consequently energy density. Research has established that Chinook stocks vary greatly in their average energy density due to differences in lipid accumulation resulting from stock specific adaptations to unique spawning locations with varying distances from the ocean, and spawning ground elevation^11,23,24^. Chinook with more difficult freshwater migrations accumulate larger amounts of lipid than those with easier ones^24^. The distinct Chinook populations distributed across the 1300 km course of the Fraser River system^25^ should therefore be expected to span a broad range of lipid content. If a SRKW can only access low quality Chinook, it will have to increase its food intake and, correspondingly, its foraging effort^26^. Williams et al.^27^ found that the amount of prey required for SRKWs could vary by a factor of 1.7 depending on the caloric estimates for Chinook salmon. With many interior and far migrating Chinook stocks (likely lipid rich) facing especially sharp declines in abundance^19^ and short migrating, hatchery Chinook (likely low lipid) proliferating in the Salish Sea in recent years, it is possible that SRKW may not be meeting their energy requirements. To evaluate this, assessment of the energy density of specific Chinook populations is needed.

Existing estimates of Chinook energy are not resolved to stock level, only discriminating between broad categories of Chinook based on spawning ground location alone—i.e., low energy coastal populations and high energy interior populations (e.g.^11^). Population specific energy density estimates are therefore an important data gap. Quantifying energy density for large salmon using traditional proximate analysis of individual whole fish is both time consuming and difficult, limiting the number of fish that can be analyzed (e.g.^11^). Fortunately, new tools exist—such as microwave energy meters (fat meters)—which can provide rapid and accurate estimates of lipid content/energy density^28–31^. Microwave fat meters measure water content and use the inverse relationship between water and lipid content to estimate lipid content^29,31^. The device can be calibrated to a species of interest and then, due to its portability, speed of use, and non-lethal nature, be used to derive lipid estimates from a much larger sample size than would be feasible with traditional methods of lipid determination. However, fat meters are designed for aquaculture and typically measure the lipid content of somatic tissue only, and since SRKWs consume whole fish, the device would need to be calibrated to this whole-body lipid values to provide ecologically useful data.

This study had the overarching objective of determining stock specific and seasonal variation in the energy density of Fraser River Chinook salmon, the primary prey source of SRKWs. To achieve this objective, we first calibrated a Distell fat meter to measure whole-body lipid content for Chinook salmon and, secondly, used this tool to quantify Chinook lipid content, and correspondingly energy density, for Chinook stocks from the Fraser River. We assess differences in lipid accumulation among Chinook populations in relation to the difficulty of their migration and discuss the potential role of the resulting differences in population energy density in the decline/stagnation of the endangered SRKW population.

## Methods

### Objective 1: calibration of fat meter

The Distell fat meter (Distell Industries Ltd, West Lothain, Scotland) is a hand-held microwave oscillator that emits a low-powered microwave that excites water molecules in an organism’s tissue at the location it is directed^30,31^. The sensor converts the measured water content into lipid content by utilizing the strong inverse relationship between water content and lipid content in fish tissue with species-specific models^11,30,31^. This tool has certain limitations. It only measures water content in the somatic tissue, ignoring the viscera, and it only measures water content in the location under the sensor, not the entire length of the fish^32^. Salmon use their fat stores from tail to front, and because salmon contain more mass in their mid-section than in their tails, it is necessary to measure the fish across the length of the body.

**Figure 1:**
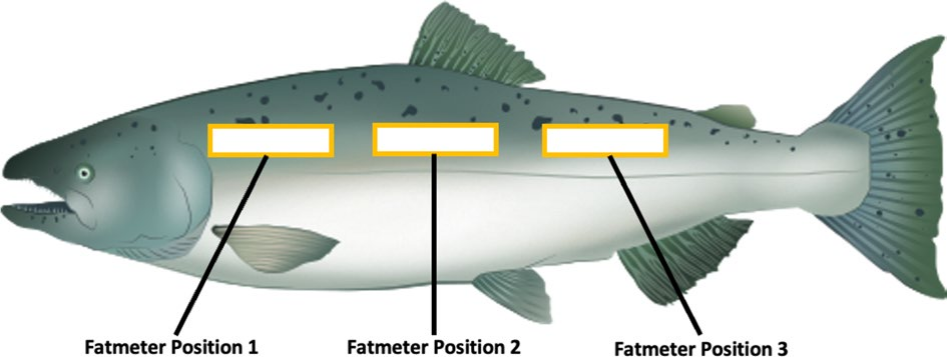
Position of fat meter readings on Chinook salmon.

The Distell fat meter can come preloaded with settings converting its measurements to, for example, Chinook fillet lipid content using a calibration from the manufacturer. There is also a ‘full-body’ Chinook calibration. As we did not know the size or source of the Chinook used these calibration, we developed our own calibration using the paired readings from the fat meter and lipid extracted, fully homogenized Chinook, including viscera and gonads^33^. To complete this calibration, we sampled across the full spectrum of Chinook salmon size and lipid content. Salmon were collected in two operations: onboard a research troller in Juan de Fuca and Johnstone Strait during the summer of 2019, and throughout the year at the Albion test fishery during the 2020 season. These salmon were both subadult salmon in the marine environment and returning adults at freshwater entry. Following the protocol of Colt and Shearer^31^ and Crossin and Hinch^30^, at collection salmon were interrogated with the fat meter in 3 locations along their anterior side, just above the lateral line (Fig. 1) using the pre-programed setting “Chinook-1”. These salmon were then euthanized, frozen and stored at − 20 °C until processing.

At processing, salmon were thawed and weighed. Following this, gonads were removed and weighed. Gonadosomatic index (GSI) was calculated as gonad mass divided by somatic mass. Carcases (plus viscera) were cut into strips and ground in an industrial meat grinder (Butcher Boy TCA32) and homogenized in an industrial food mixer. Gonads were homogenized in a commercial blender. Water and lipid content were determined gravimetrically. For lipid extractions, three separate 7–10 g subsamples were taken from different portions of the homogenized material. These samples were then dried at 50 °C for 72 h and water content was determined. Dried material was ground using a mortar and pestle. Lipid content was determined for both the somatic and gonadal tissue using a 2:1 chloroform:methanol extraction following Folch et al.^34^ as revised by Post and Parkinson^35^ and Arrington et al.^36^. Somatic samples were processed in triplicate and gonad samples were processed in duplicate, with means reported. Somatic and gonadal lipid measurements were converted back to wet weight and combined to produce whole-body lipid content.

### Statistics

Fat meter readings at each location (and the mean of every possible combination thereof) were regressed against the calculated whole-body lipid content. Three different regression were attempted. As both the fat meter values and the lipid content can be considered dependent variables with some error, the first was a simple model II linear regression performed using the raw fat meter values and measured whole-body lipid content. As both Colt and Shearer^31^ and Crossin and Hinch^30^ used log_e_ transformed fat meter values in their analyses, a second model II linear regression was performed using log_e_ transformed raw fat meter values and measured whole-body lipid content. Finally, because the fat meter can saturate at low lipid values and deviate from its calibrated linear lipid-water relationship, a segmented regression (r package segmented) using the raw fat meter values and measured whole-body lipid content was performed. The model with the greatest coefficient of determination (r^2^) was selected. All analysis was done is R version 5.0 (R core team). To determine if sex impacted the relationship to the fat meter calibration, we compared sloped and intercepts of regressions for these variables using Analysis of Covariance (ANCOVA). To determine the effect of size (kg) and GSI on the calibration we determined the interaction of each of these variables and the fat meter values in the best performing linear regression model.

### Objective 2: measurement of Fraser Chinook lipid levels

**Figure 2:**
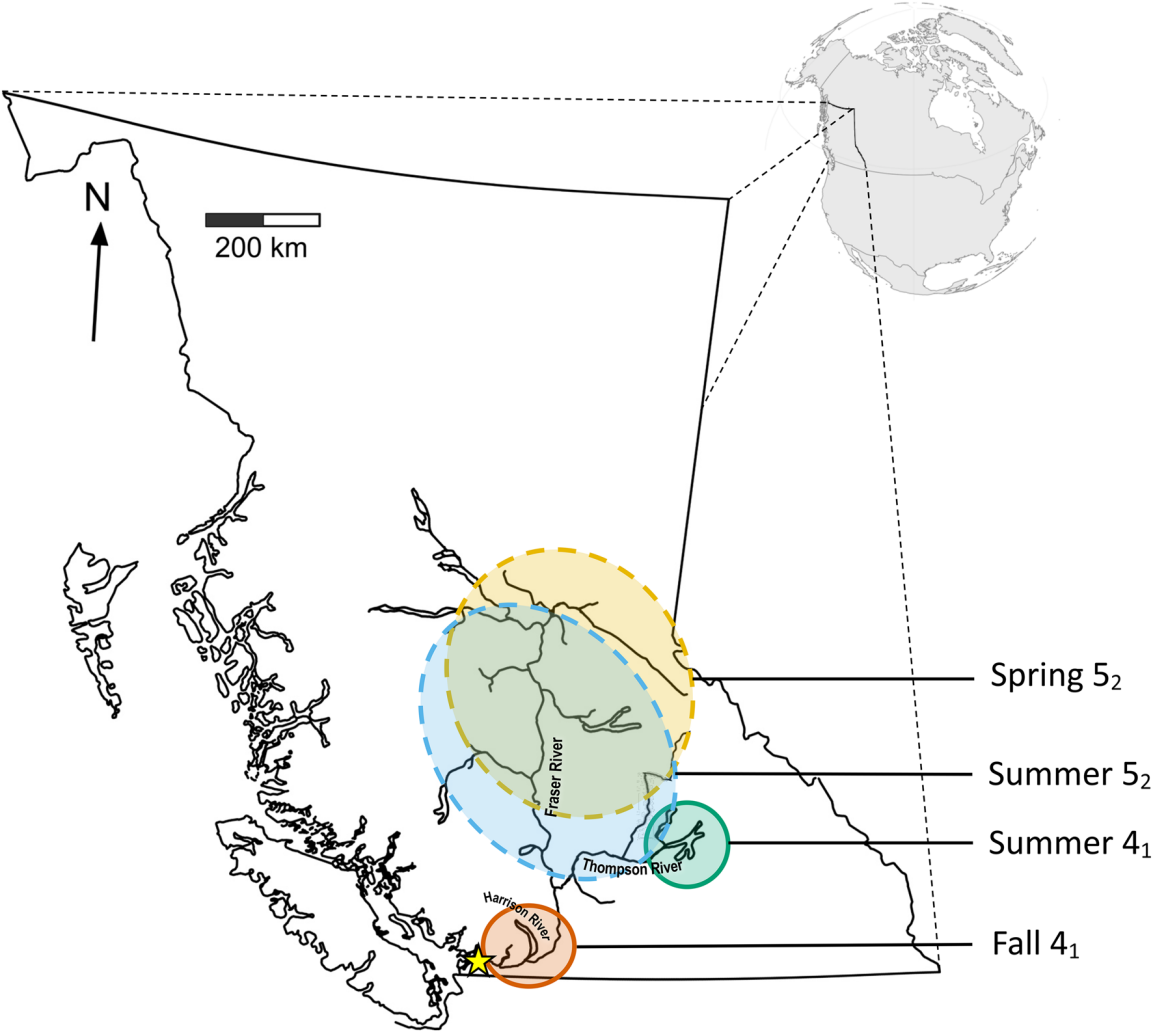
Map of Fraser River watershed showing location of Albion test fishery (yellow star) and distribution of the four main Chinook management units (colored circles). Dashed circles indicate the more diffuse spawning regions of the Spring 5_2_ and Summer 5_2_ management units.

During the summer of 2020, a Distell fat meter was deployed at the Albion Chinook test fishery. The Fisheries and Oceans Canada (DFO) operated Albion Chinook test fishery is conducted on the lower Fraser River at Albion, BC (Fig. 2). from early April until mid-October. The test fishery is conducted with a drifted gill net. There are two test sets a day, timed to coincide with the daily high tide. The fishery uses two different nets which fish on alternating days: the "standard" Chinook net, which is constructed using eight-inch mesh; and a "multi-panel" net, which consists of panels of six, seven, eight, and nine inch mesh. The purpose of the multi-panel net is to ensure representative sampling of Chinook passing through the lower Fraser River, due to the wide range of body sizes observed in Fraser River Chinook stocks. Both gill nets used in the Albion Chinook test fishery are 365 m long. From September 1st through October 20th, the Albion Chinook test fishery operates every other day and uses the eight-inch mesh net exclusively.

The test fishery intercepts Chinook from all major Fraser River populations. Fishery managers divide the Fraser River Chinook populations into five management units (MUs). These management units are groupings of conservation units^37^ grouped by shared life history traits: run-timing (spring, summer, fall), average age of return (typically year 4 or 5) and early life history (stream-type or ocean-type, described as years spent in freshwater, 2 or 1 year respectively). In the Gilbert-Rich aging designation system, the five main MUs are denoted: Spring 5_2_, Spring 4_2_, Summer 5_2_, Summer 4_1_ and Fall 4_1_^38^. As many of these life history traits are driven by climatic variables^39^ these MUs have occupy broadly distinct geographic ranges^25^ with the Fall 4_1_ Chinook found at the lowest reaches of the Fraser centered on the Harrison and Chilliwack Rivers, the Summer 4_1_ Chinook mid-way up the Fraser in the South Thompson River watershed, and the Spring 5_2_, Spring 4_2_ and Summer 5_2_ in overlapping ranges in the upper reaches of the Fraser or North Thompson Rivers with the Spring 5_2_ occupying the most upstream systems in the Rocky Mountain foothills (Fig. 2). There are a few exceptions to these geographic distributions, for example, the Upper Pitt River summer run conservation unit is included in the Summer 5_2_ MU despite being geographically located in the lower Fraser while the bulk of the MU is found in the middle Fraser. In those exceptional cases, our analysis removed individuals from unique populations far outside the geographic range of the majority of their MU.

Test fishery crew annually collect biological data including weight, fork length as well as a scale for genetic stock ID for all Chinook collected. Genetic stock assignments were performed at the Molecular Genetics Laboratory (Pacific Biological Station, Fisheries and Oceans Canada), using cBAYES and a reference baseline derived from microsatellite markers that consisted of 268-populations with more than 50,000 individuals^40^. The crew was instructed on operation of the fat meter and performed/recorded three measurements on all Chinook encountered in addition to their normal measurements. The fat meter was deployed for the entire 2020 Albion test fishery season except for August 15th-August 18th when it was inactive due to a minor malfunction.

### Difficulty of Chinook migration

Following the protocol of Crossin et al.^41^, two environmental variables associated with the difficulty of Chinook migration were measured. These were migratory distance and elevation of spawning ground. Because these variables can be cross-correlated, combinations of them, namely work (distance × elevation) and river slope (500 × (elevation/distance), were also assessed. Data for these variables was collected from the literature for the specific stocks that constituted the Fraser River Chinook MUs analyzed in this study (Table 1), and then averaged for each MU.

**Table 1:** Four indices of migration difficulty for Fraser Chinook salmon.

	Spring 5_2_	Spring 4_2_	Summer 5_2_	Summer 4_1_	Fall 4_1_
Mean elevation (m)	713	570	606	350	93
Mean distance (km)	994	392	705	523	112
Mean work	712.9	223.6	464.9	186.5	11.3
Mean slope	375.3	726.2	426.1	341.9	409.3

Mean elevation (m) and distance from freshwater entry (km) of spawning ground, work [(elevation × distance)/1000] and slope (elevation/distance) × 500. Data used to determine elevation and migration distance taken from the literature^64–70^.

### Statistics

The calculated regression from objective 1 was used to correct fat meter measurements for whole-body lipid content. Energy density was calculated using the equation provided by O’Neill et al.^11^ relating Chinook whole-body lipid content to kcal/kg. Analysis of Variance (ANOVA) plus Tukey tests were performed to compare average lipid content between stock groupings and sex/hatchery influence within stock groupings. To provide context on differences in MU-specific run timing, we plotted average weekly MU-specific catch-per-unit-effort (CPUE), where effort is fathom-minutes per boat day, at the Albion test fishery between 2000 and 2014. See MacDonald^42^ for the details of the CPUE calculations.

To assess differences between wild and hatchery reared salmon, we recognized that on the Fraser River salmon hatcheries release individuals from several MUs but the largest number of hatchery releases are from the Fall 4_1_ MU. Hatchery released individuals typically have their caudal fin clipped to mark them as non-wild salmon. To assess the difference between hatchery and wild Chinook, we performed a t-test on fin-clipped individuals and non-fin-clipped individuals from a single population within this MU, the Harrison River population. The Harrison River is a tributary of the Fraser River and a major Chinook spawning ground for the Fall 4_1_ MU. The Chinook population that spawns there was selected, rather than the more general Fall 4_1_ MU, as it is home to both one of the largest wild Chinook runs on the Fraser and a Chinook hatchery where 100% of Chinook are fin clipped. Not all Chinook from other Fall 4_1_ MU hatcheries are fin clipped, so a more narrow focus on the Harrison River system allowed us to isolate wild/hatchery interactions.

Linear regression was used to determine effect of day of arrival, weight, and fork length on fat content within groupings and, when applicable, ANCOVA was used to compare this effect between groups. Linear regression was also used to determine the effect of day of arrival on male and female gonad lipid content. We calculated Pearson’s correlation coefficients to evaluate the relationship between the four indices of Chinook migration and mean whole-body lipid content at the Albion test fishery for the five major Fraser River MUs.

#### Animal care

This study was carried out under the animal care protocol approved by the University of British Columbia Animal Care Committee (UBC Animal Care Protocol #A18-0037) in accordance with CCAC guidelines (The Care and Use of Fish in Research, Teaching and Testing, 2005) and Fisheries and Oceans Canada’s national guidelines. Reporting in the manuscript follows the recommendations of the ARRIVE guidelines.

## Results

### Fat meter calibration

A total of 63 whole Chinook were collected, 21 from the troll in 2019 and 42 from the test fishery in 2020, for homogenization and proximate analysis. These fish ranged from < 0.25 kg to 11.05 kg with a fork length of 26.5 cm to 76.7 cm. They spanned raw fat meter measurements (as measured from position 1) of 0.6 to 16.8. GSI ranged from 21.4 in a large female sample to < 1.0 in the immature marine samples (Fig. S1). Lipid content (% wet weight) of female gonads averaged 10.03% ± 1.89 while male gonads averaged 0.68 ± 0.36. Lipid content of both male and female gonads decreased over the season, but this relationship was not significant for either sex (linear regression, p > 0.05 for both sexes).

**Figure 3:**
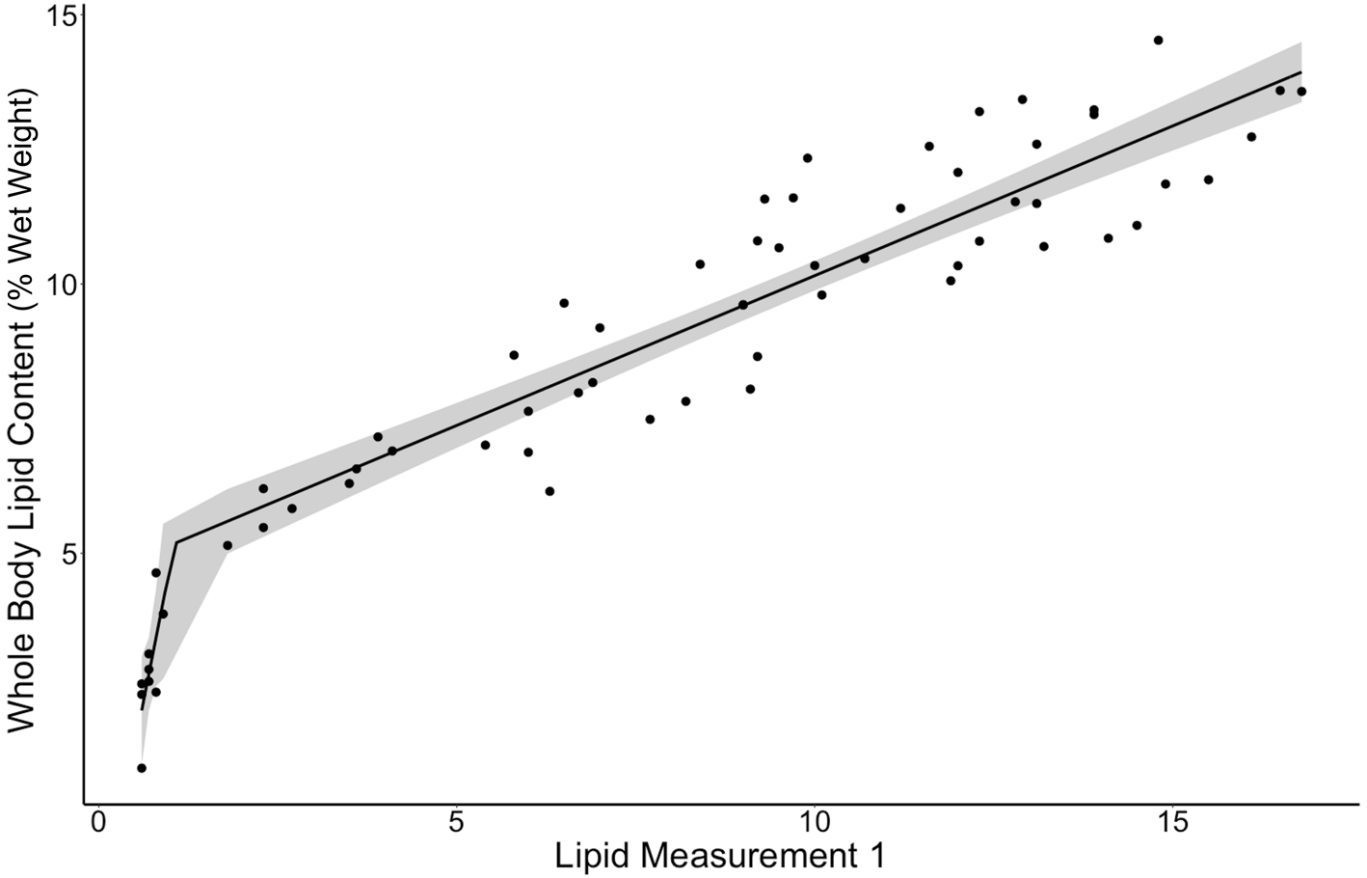
Segmented regression between raw lipid measurements at fat meter position 1 and whole-body lipid content (% wet weight) (r^2^ = 0.91). Gray shading denotes 95% confidence interval.

Regression of whole-body lipid content and fat meter readings recorded strong coefficients of determination (r^2^ > 0.82, p < 0.001) regardless of the position or combination thereof used, and regardless of whether the data were raw or log_e_ transformed or the regression a model II or segmented regression (Table 2). The top coefficient of determination was generated by the segmented regression model using raw fat meter measurements from fat meter position 1 (r^2^ = 0.91, breakpoint: x = 1.059, residual standard error = 1.03) (Fig. 3). We therefore used the equation produced by this model in the subsequent calculation of whole-body lipid content in Chinook salmon:

**Table 2:** Coefficient of determination (adjusted R^2^) values for segmented and model II regressions between whole-body lipid content (% wet weight) and raw and log_e_ transformed fat meter values from individual positions and averages of all combinations of positions.

Position	Model II	Model II Log	Segmented
1	0.881	0.875	0.914*
2	0.831	0.867	0.883
3	0.796	0.863	0.869
1, 2	0.874	0.879	0.911
1, 3	0.864	0.886	0.909
2, 3	0.829	0.874	0.890
1, 2, 3	0.869	0.886	0.911

Where Fat Meter Reading < 1.059: Whole-Body Lipid Content = − 1.973 + 6.758 × Fat Meter Reading;

Where Fat Meter Reading > 1.059, Whole-Body Lipid Content = − 1.973 + 6.758 × 1.059 + ((6.758 ± 6.202) × (Fat Meter Reading − 1.059)).

We observed no effect of sex on the regression equation (ANCOVA, p > 0.05) and therefore our calibration was applied equally to male and female Chinook salmon. We also observed no significant interaction between the fat meter values and either GSI or size (kg) in the regression with whole-body lipid content (linear regression, p > 0.05).

### Measurement of priority Chinook stocks lipid levels

**Figure 4:**
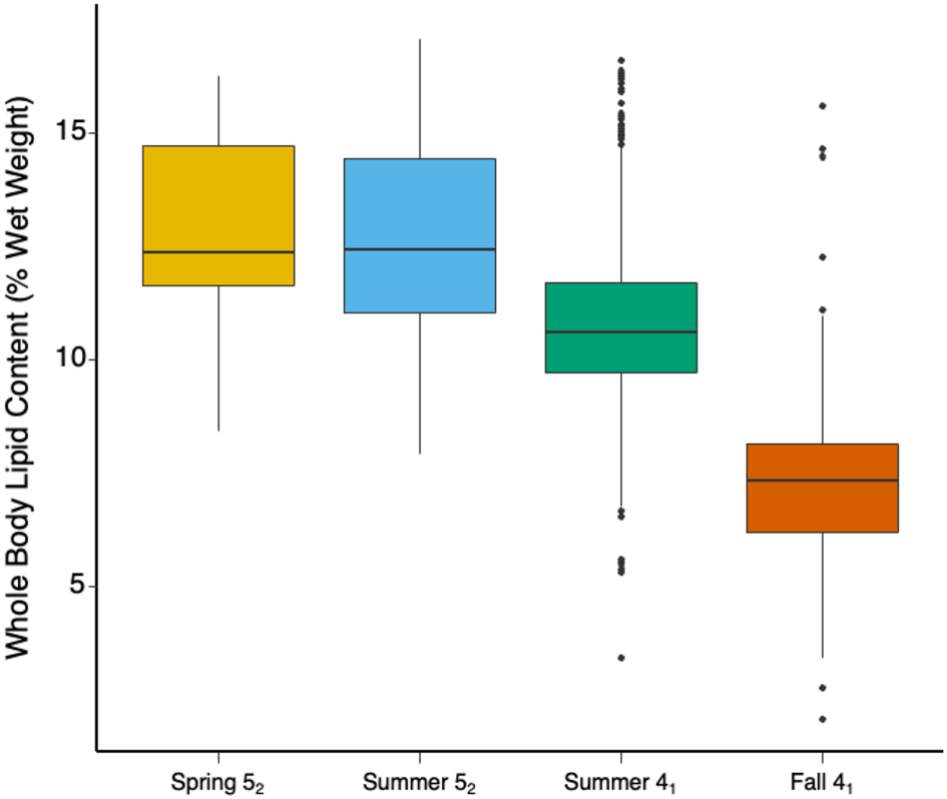
Boxplot of Fraser River management unit whole-body lipid content. The black bar represents the mean lipid level. The upper and lower hinges correspond to the 1st and 3rd quartiles (25th and 75th percentiles). Whiskers extends from the hinge to the largest value no further than 1.5 × IQR from the hinge (where IQR is the inter-quartile range, or distance between the first and third quartiles). Data beyond the end of the whiskers are outlying points and are plotted individually.

Biological measurements were collected on a total of 1566 Chinook individuals at the Albion test fishery in 2020. The majority of these samples (n = 966) came from the Summer 4_1_ MU. The Spring 4_2_ MU had the smallest sample (n = 9) and was therefore not included in statistical analyses. Average MU specific whole-body lipid content, energy density, fork length and weight are reported in Table 2. Calculated whole-body lipid content differed significantly among MUs (ANOVA, p < 0.001) and all MUs differed significantly from one another (Tukey’s HSD, p < 0.001) except for Spring 5_2_ and Summer 5_2_ (Table 3, Fig. 4). Lipid content (% wet weight) was highest in the Spring 5_2_ (12.8 ± 2.3) and Summer 5_2_ MUs (12.7 ± 2.3), intermediate in the Summer 4_1_ MU (10.8 ± 1.8), and lowest in the Fall 4_1_ MU (7.3 ± 1.6). Average fork length (mm) was > 600 mm for Spring 5_2_ (660.9 ± 67.4), Summer 5_2_ (658.3 ± 55.0), Summer 4_1_ (638.2 ± 49.8) and Fall 4_1_ (671.5 ± 61.4), and < 600 mm for Spring 4_2_ (584.7 ± 70.9). Mean weight (kg) was highest in Fall 4_1_ (8.2 ± 2.6) and lowest in for Spring 4_2_ (4.8 ± 2.1).

**Table 3:** Mean ± standard deviation, min, max, and sample sizes for calculated whole-body lipid content (% wet weight) for Fraser River Chinook Management Units measured at the Albion test fishery in 2020.

	Spring 5_2_	Spring 4_2_	Summer 5_2_	Summer 4_1_	Fall 4_1_
Sample size	28	9	72	966	473
Lipid content (%)					
Mean	12.8 ± 2.3^A^	12.2 ± 1.7	12.7 ± 2.3^A^	10.8 ± 1.8^B^	7.3 ± 1.6^C^
Min	8.4	10.7	7.9	3.4	2.1
Max	16.3	16.3	17.1	19.8	15.6
Energy density (kcal/kg)					
Mean	1900 ± 194	1849 ± 147	1890 ± 196	1728 ± 151	1436 ± 138
Min	1539	1720	1487	1101	985
Max	2200	2205	2271	2229	2143
Fork length (mm)					
Mean	660.9 ± 67.4^AB^	584.7 ± 70.9	658.3 ± 55.0^A^	638.2 ± 49.8^B^	671.5 ± 61.4^A^
Min	507	458	484	426	493
Max	752	734	751	826	865
Weight (kg)					
Mean	7.0 ± 2.4^AB^	4.8 ± 2.1	7.1 ± 1.9^A^	6.4 ± 1.6^B^	8.2 ± 2.6^C^
Min	2.7	3.2	2.7	2.3	3.6
Max	11.3	10.4	11.8	13.2	17.7
Run timing 2020					
First	2020-04-21	2020-04-22	2020-07-02	2020-07-16	2020-08-24
50%	2020-07-13	2020-07-15	2020-08-13	2020-08-28	2020-09-21
Last	2020-09-07	2020-08-02	2020-09-04	2020-09-29	2020-10-27

Significant differences denoted by different letters (due to small sample size Spring 4_2_ not included in analyses).

There was no significant difference between male and female whole-body lipid content within MUs (Tukey’s HSD, p > 0.05). Likewise, we observed no significant relationship between weight or fork length on whole-body lipid content within MUs (linear regression, p > 0.05 for all MUs) (Table S2). We did observe a significant relationship between day of arrival and whole-body lipid content for all MUs (linear regression, p < 0.001) (Table S2).

**Figure 5:**
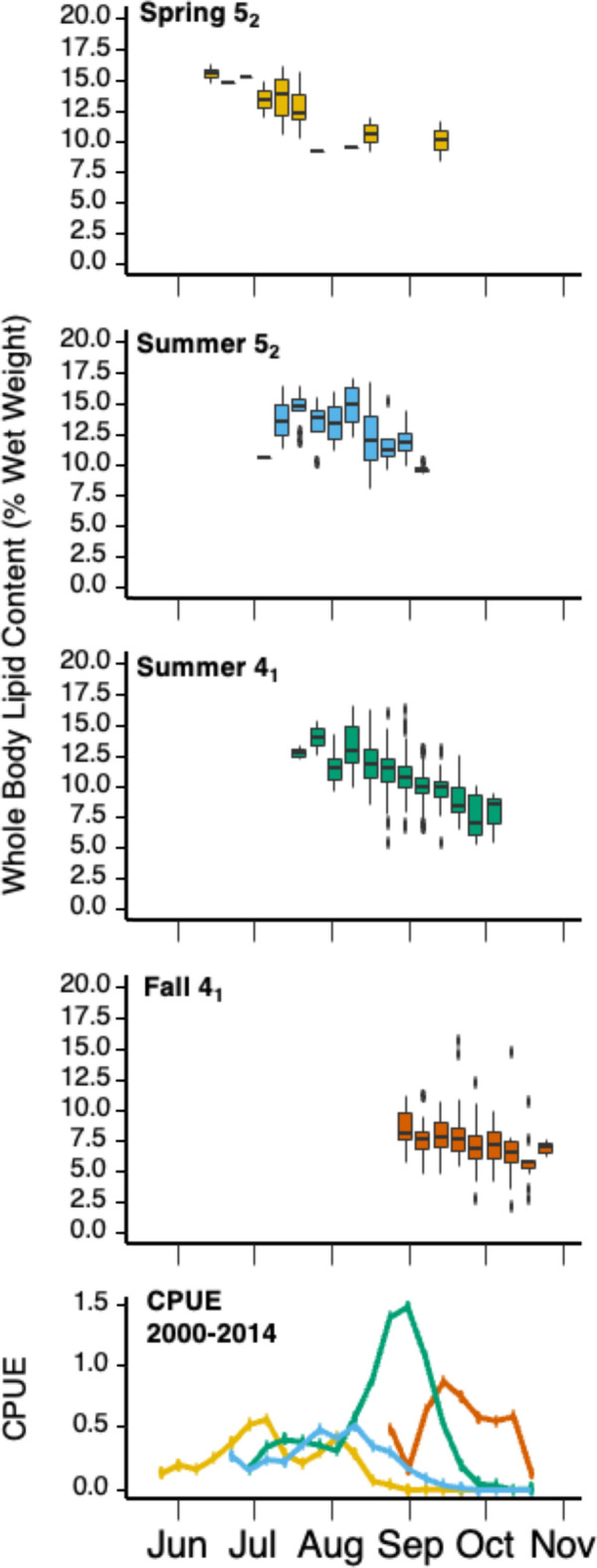
Boxplot of weekly whole-body lipid content by management unit. Black points in the boxplots represent mean lipid content. The upper and lower hinges correspond to the 1st and 3rd quartiles (25th and 75th percentiles). Whiskers extends from the hinge to the largest value no further than 1.5 × IQR from the hinge. Data beyond the end of the whiskers are outlying points and are plotted individually. Bottom plot illustrates weekly mean CPUE for each Fraser River Chinook MU at the Albion test fishery from 2000 to 2014.

The CPUE measurements taken by MacDonald^42^ illustrated the expected^43^ distribution of MU arrival time at the Fraser River, with the Spring 5_2_ arriving first, followed by the Summer 5_2_, the Summer 4_1_ and finally the Fall 4_1_ (Fig. 5). Lipid content declined over the migration period for each MU, by ~ 5 percentage points (pp) for Spring 5_2_, ~ 5pp for Summer 5_2_, 6pp for Summer 4_1_, and ~ 4pp for Fall 4_1_ (Fig. 5). The MU specific slopes of this relationship were similar, except for the slopes of both the Summer 5_2_ and Summer 4_1_ being significantly steeper than Fall 4_1_ (ANCOVA, p < 0.001). For Harrison River Chinook, the lipid content of fin-clipped individuals and non-fin-clipped individuals was 6.48%% and 7.30% respectively and there was no significant difference between the two groups (t-test, p = 0.22).

**Figure 6:**
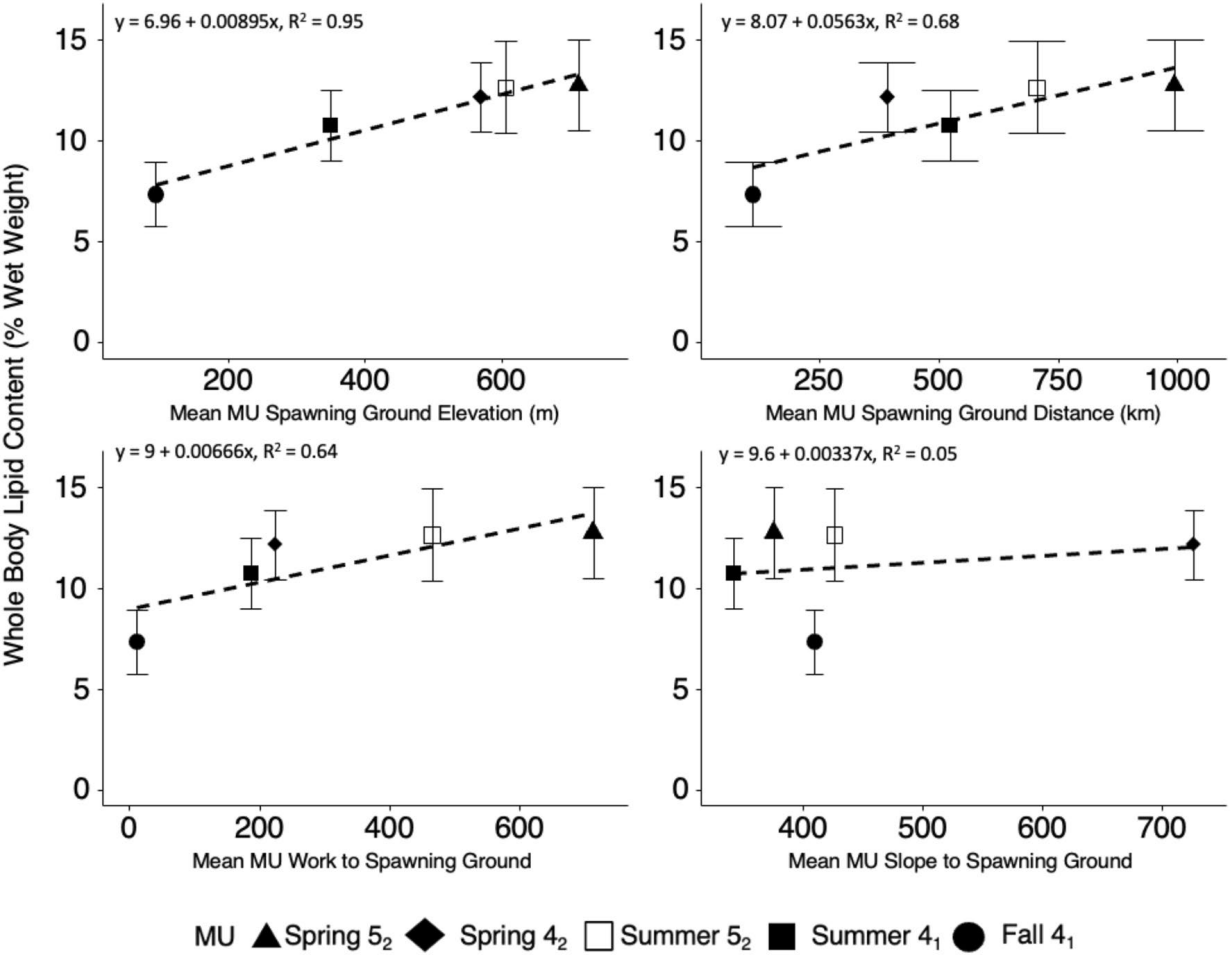
Relationship between four indices of migration difficulty (spawning ground elevation, spawning ground distance, work to spawning ground, slope of spawning ground) and mean (± SD) whole-body lipid content for the five Fraser River Chinook MUs. Linear regressions are fitted to the data.

A review of difficulty of Chinook migration revealed that Spring 5_2_ Chinook had the longest average migrations followed by the Summer 5_2_, the Summer 4_1_, the Spring 4_2_, and last the Fall 4_1_ Chinook populations (Table 1). Spring 5_2_ Chinook also spawned at the highest elevation followed by the Summer 5_2_, the Spring 4_2_, the Summer 4_1_ and finally the Fall 4_1_ Chinook populations (Table 1). Pearson’s correlation coefficients for the four indices of Chinook migration difficulty and MU mean whole-body lipid content at the Albion test fishery were 0.97 for mean spawning ground elevation (p = 0.005), 0.82 for mean distance (p = 0.088), 0.80 for mean work (p = 0.10) and 0.23 for mean slope (p = 0.71) (Fig. 6).

## Discussion

Resident killer whales off the Pacific coast of Canada are fish specialists with a high reliance on large, coastally distributed Chinook salmon as prey. In this study we examined life history variation in Fraser River Chinook lipid content, measured with a microwave fat meter, as a potential factor in the population decline/stagnation of SRKWs. To properly interpret our results, we first comment on the successful calibration of the fat meter to Chinook salmon to establish its efficacy. We then review the results of the deployment of the fat meter to the Albion test fishery and the stock specific lipid content data with regard to the different Chinook life histories present in the Fraser River and their difficulty of migration. Having delimitated Fraser River Chinook lipid levels with respect to stock and seasonal variability, we address the implications for the prey quality experienced by SRKWs and their ability to meet their nutritional requirements.

### Fat meter calibration

Our results confirmed that the handheld microwave fat meter can serve as an effective tool for rapid measurement of the lipid content of Chinook salmon. This result corroborates research on both sockeye^30^ and Columbia River Chinook populations^31^ that found the fat meter device successful at determining somatic lipid content. The calculated regression showed the fat meter was effective at predicting whole-body lipid content in Chinook with a residual standard error of 1.03.

Lipid content was measured at three locations on the fish, yet our analysis indicated the best relationship between the measurements and the true whole-body lipid content required only the measurements at position 1. This is likely because Chinook use energy from the tail to the front, and the front of the organism contains a large portion of their mass. Raw fat meter measurements appeared to reach a nadir at 0.6 and were never recorded lower. Chinook samples with fat meter values near 0.6 had a whole-body lipid content of roughly ~ 2%. Though this may represent the lowest fat value the fat meter is capable measuring, it also intersects the lowest level of lipids a salmon can maintain^41^.

Our analysis was performed to determine whole-body lipid content. To do so, we included lipids from both the viscera and gonads. The fat meter could not have reasonably measured lipids in the gonads and viscera as it only penetrates 1–3 cm into the flesh^31^, so any variability caused by lipid levels in these tissues had to be captured in the final regression. Viscera contains only a small amount of lipids^44^ and therefore the impact of viscera lipid on whole-body lipid level was minimal. More noteworthy was the lack of an impact of sex on the relationship. Despite both known^44^ and observed differences (Table S1) in male and female gonad lipid content, there was no significant difference in the regression between male or female fish. We also found no effect of GSI on the calibration. This could be because all salmon sampled were either captured in the marine environment (immature) or at freshwater entry with (low levels of sexual maturity) so that they lacked gonad investment on a scale that could impact the calibration. Due to differences in spawning time and holding periods, Chinook at freshwater entry arrive at different levels of maturity^45^. The individuals sampled for our regression had gonads that encompassed 1–17% of total body mass. However, only females arrived with large gonads (> 10% of body mass) and those that did were entirely from the later arriving, Fall 4_1_ MU. These Fall 4_1_ Chinook had an average lipid content of 7.34%. Because Chinook ovaries had an average lipid content of ~ 10%, their impact on whole-body lipid content was minimal as this percentage was not too high or low to drastically change whole-body lipid content in female salmon with large gonads. Ovary lipid content drops as Chinook approach spawning^44^. Therefore, the fat meter is capable of providing accurate whole-body lipid content estimates of both male and female Fraser Chinook provided they are either immature or caught at river entry.

The ability to rapidly catalogue lipid content in Chinook is important for assessing SRKW prey requirements, but beyond this application it has significant potential to inform Chinook ecology. Lipid content in fish can be correlated with ocean conditions^46^, the physiological status of fish^47^, salmon migrations^48^, and the threat of pre-spawn mortality^49^. The successful calibration of this tool should create research opportunities for Chinook both in the marine environment and at freshwater entry.

### Stock specific Chinook salmon lipid content

Applying the calibrated fat meter we were able to quantify and catalogue the lipid content of Fraser Chinook salmon stocks at river entry. Based on mean average lipid content, we identified three distinct groups from the Fraser MUs. These groups were (1) High: Spring 5_2_ and Summer 5_2_ (12.7%), (2) Intermediate: Summer 4_1_ (10.8%), and (3) Low: Fall 4_1_ (7.3%). It is recognized that Chinook salmon populations with more arduous migrations will have greater energy content^23^ and we observed the same pattern in the results of our analysis of indices of migration. With only five data points (including the small Spring 4_2_ MUs), our Pearson’s correlation analysis had limited power, but our results showed that whole body lipid content was positively correlated with all four indices of migration difficulty. Spawning ground elevation had the strongest relationship to whole body lipid content, though mean distance and work also had strong correlations. In our analysis, Spring 5_2_ and Summer 5_2_ Chinook had the highest lipid levels. These two MUs have some of the longest migrations on the Fraser River (995 km and 705 km respectively), coinciding with greater elevation of spawning ground sites (712 m and 605 m).

The highest whole-body lipid content values for the Spring 5_2_ and Summer 5_2_ MUs lipid grouping were similar to those obtained near river entry from previous whole-body analysis of far-migrating populations, such as spring Chinook stocks from the lower Columbia (14.8%) and Chena (tributary of the Yukon) (15.9%) rivers^11,28^. These values potentially capture the high end of Chinook lipid accumulation coast wide. The mean lipid content values for the Spring 5_2_ and Summer 5_2_ MUs were slightly lower than the values from the aforementioned river systems but there are several potential reasons for this. First, the Chena Chinook have more than twice as long a migration as Fraser Spring 5_2_ Chinook, though they gain half the elevation. Second, while the lower Columbia River Chinook migrations are neither as long nor gain as much elevation as the Fraser Spring 5_2_ and Summer 5_2_ MU, our values may underestimate these Fraser stocks lipid content due to measurements primarily coming from late in their run cycle.. We identified a trend within all Fraser MUs that more lipid rich Chinook arrived earlier. Because the Albion test fishery has struggled to catch the earlier portions of these runs, both due to high early-season river flows and depressed upriver Chinook populations, we may have missed some of the fattier Chinook from the Spring 5_2_ and Summer 5_2_ populations. Conversely, because both the Columbia and Yukon river studies sampled individuals over only a few days near the beginning of their respective runs, they may have missed some of the lower lipid, later arriving individuals in their analyses.

The Summer 4_1_ stock had the second highest average lipid content of the Fraser River Chinook runs. Though migrating later than the Summer 5_2_ MU, the Summer 4_1_ population spawns ~ 525 km upriver at ~ 350 m elevation and their high lipid content likely reflects the still large elevation and distance that they need to cover. Values for this stock were lower than those calculated for a “Fraser population complex” (consisting of a majority Summer 4_1_ individuals) from O’Neill et al.^11^. Our analysis sampled Summer 4_1_ fish over the course of their migration from August through September, while the O’Neill et al.^11^ analysis sampled over one week in August. Since Summer 4_1_ MU-specific lipid levels dropped over the course of season, as with other stocks, the August timing of the former study may have led to an inflated estimate of lipid content.

The final MU, the Fall 4_1_ had the lowest lipid content at 7.34%. This low lipid content is likely driven by the short migrations for these stocks. The Fall 4_1_ MU consists mainly of the Harrison River stock which migrates only 110 km and 12 m elevation to its spawning ground. As with the other Fraser MUs, there was a wide range in lipid values of returning Fall 4_1_ Chinook, but this range included Chinook with whole-body lipid content of < 3%. This would suggest that some Chinook from this MU are arriving from sea with lipid levels near the lowest possible to maintain life and questions the potential spawning succuss of those individuals^49^. The Fall 4_1_ MU had the largest weight and fork length, corroborating previous research that Chinook with shorter migrations tend to be larger than those with longer ones^50^.

As mentioned earlier, an interesting trend identified by this analysis was that within Fraser MUs, more lipid-rich fish from every population tended to arrive earlier. The difference was most pronounced in the Summer 4_1_ MU and least in the Fall 4_1_ MU. It is possible this is a result of increased gonadal investment among individuals arriving later relative to earlier in the run. GSI did increase over the course of the run for both Summer 4_1_ and Fall 4_1_ Chinook (Fig. S1), so this potentially played a role in the observed trend, however GSI did not increase enough to cause as large a decrease in whole-body lipid as we observed. To take the most extreme example: an average sized Fall 4_1_ Chinook (8 kg) had a GSI increase from 0.1 early in the run to 0.2 late (the largest GSI increase observed in an MU in our study) and whole-body lipid percent decrease from 8.4% during the first week of the run to 5.4% lipid during the second to last (the lowest across season lipid decrease observed in an MU in our study). The amount of energy required for the level of gonadal development measured (assuming gonad energy density of 7.79 kJ/g^51^) would be at least 4720 kJ. Using the equation converting whole-body lipid content to energy density from O’Neill et al.^11^, the difference in whole-body energy between the first week of the run and the second to last equals 8600 kJ. If the sole reason for the lower lipid values of later arriving Chinook was increased gonadal investment, we should not expect to see as large a difference in lipids we observed. Our results therefore appear to capture a true effect of earlier arriving salmon having higher lipid content. Earlier arriving fish tend to live longer on the spawning grounds than late arriving fish^23,51^. Whether the higher lipid levels observed in early spawners are a cause or an adaptation to this behavior cannot be determined at this time. Earlier arriving Chinook may also have hit an energy threshold triggering their return to the spawning ground earlier in the season, whereas late arriving Chinook may be balancing returning with lower lipid levels in the current spawning season with the risk of predation inherent in another season at sea before maturity^23^.

### Implications for SRKWs

The Chinook lipid content values identified in this study have important implications for understanding SRKW prey requirements. To begin, our study has identified greater diversity in the lipid content expressed by Chinook salmon stocks than previously proposed. Previous research has delimited salmon lipid content between two groups: low fat (2–5% of somatic tissue), coastal populations and high fat (12–15% of somatic tissue), interior-spawning populations^11,24^. Though we also identified distinct high and low lipid content populations, here we illustrate a major Chinook population with lipid levels intermediate between these two groups—the Summer 4_1_ MU (10.8%).

Our MU specific estimates of Chinook lipid content will allow for more accurate predictions of the needs of the SRKW population. Energy density in salmon is primarily driven by lipid content^30^ and O’Neill et al.^11^ found a very strong (r^2^ = 0.97) relationship between whole-body lipid content and energy density. Using this relationship to determine mean energy density (see Table 2), the energetics model established by Williams et al.^27^ and assuming an average 8 kg Chinook prey, the SRKW population consuming only Spring 5_2_ Chinook would require 245,000 Chinook/year whereas the population consuming only Fall 4_1_ would require 325,000 Chinook/year, a difference of 80,000 salmon. Those calculations are, of course, unrealistic; SRKWs do not spend the entire year foraging in the Salish Sea, nor do they forage on one population of Chinook exclusively However, they do illustrate how the MU specific energy data can be particularly powerful when combined with high resolution stock specific diet information such as compiled by Hanson and colleagues^17,52^. We note, however, that the magnitude of the within-MU decreases in lipid content across a season (such as the ~ 6pp drop in whole-body lipid content observed in the Summer 4_1_ MU from July to September) reveals that a more nuanced temporal approach to population specific estimates of energy density may be warranted.

SRKWs spend their summer in the Salish Sea, foraging predominately on the major Fraser River runs as they return^17^. Critically, due to population-specific marine distributions^53^ and run timings^25^, not all these Chinook populations are available to SRKWs in the Salish Sea at any given time. As this study shows, as the season develops and the later season runs arrive, the energy density of SRKWs’ Chinook prey declines. All Spring and Summer Fraser Chinook MUs, which are more valuable in terms of calories, are only available as prey for SRKWs for a short window of time because these stocks are considered offshore or far-north migrating and cannot be found year-round in the Salish Sea^19^. Instead they are only accessible in this habitat during their return migrations in June–September.

Historically, SRKWs have tended to arrive in the Salish Sea in May/June to prey on the most lipid-rich Spring 5_2_ and Summer 5_2_ MUs^54^. These MUs have generally been in decline since the early 2000s, potentially depriving SRKWs of a critical energy source^19^. In fact, in recent year SRKWs have been spending less time in the region during the spring months with the reason believed to be low abundances of Spring 5_2_ and Summer 5_2_ Fraser River Chinook^54^. SRKWs likely now instead spend this time of year near the Columbia River^2^ where they can intercept interior Columbia Chinook which, due the similarity of their long migrations to Spring 5_2_ and Summer 5_2_ Fraser Chinook MUs, should be energy rich^52^. The whales may be better served waiting to arrive in the Salish Sea until the larger and intermediate lipid-rich Summer 4_1_ run arrives in July^19,55^. However, as our data also demonstrated a large drop in average MU-specific lipid levels over the course of the season, any delay in SRKW arrival comes at the cost of missing higher value Fraser Chinook prey.

As we have shown, the Summer 4_1_ run represents a unique intermediate energy prey source for SRKWs. When the Summer 4_1_ run tapers out in September, SRKWs switch to Fall 4_1_ and, eventually, Puget Sound Chinook. These stocks are considered ‘local’ and thus express the year-round availability trait proposed as one of the reasons Chinook are so valuable to SRKWs^19^. The importance of this year-round availability is evident as both Puget Sound and Fraser Fall 4_1_ Chinook constitute a significant portion of SRKW diet throughout the winter when there are no returning populations^52^. However, these salmon have a low energy density. Our data would predict that Puget Sound Chinook would share the low lipid content of Fraser Fall 4_1_ Chinook as they share similar life history strategies, run-timings, and short migrations. All else being equal, SRKWs would need to consume more of these Fall 4_1_ Puget Sound Chinook than any of the Spring/Summer populations to get the same amount of energy. However, because these Fraser Fall 4_1_ and Puget Sound populations are more numerous they may be easier to access^18,19^. Additionally, our data indicated Fall 4_1_ Chinook were larger than Spring/Summer Chinook. The larger size of the Fall 4_1_ Chinook could compensate for the lower energy density of this population, although, as hatchery raised Chinook tend to be smaller than wild ones^50^, this effect may be attenuated by the prevalence of hatchery-influenced stocks in fall-run Chinook populations.

Both Fraser Fall 4_1_ and Puget Sound origin Chinook populations have significant hatchery influence. In fact, outside of the Spring/Summer Fraser runs, hatchery-influenced stocks form the base of most SRKW priority Chinook populations^17,52,56^. In our study the difference in lipid content between hatchery and wild fish was not significant, but lipid content in the former was 0.82% lower. By using only Harrison River Chinook, where there is a large wild population and a small hatchery with 100% visual marking^57^, we attempted to control for hatchery influence. However, our results may be confounded by the genetically similar Harrison-origin Chinook produced at the Chilliwack River hatchery 40 km from the mouth of the Harrison River, not all of which are fin clipped. This means that some individuals included in the ‘wild’ fish group could have been reared at the Chilliwack hatchery. The difference of 0.82% that we observed may therefore be conservative, but still represented a difference in energy density of 293 kJ/kg. Differences between lipid levels of wild and hatchery salmonids have been observed in steelhead, *Oncorhynchus mykiss*^58^, and the evolutionary mechanisms responsible for this difference, such as the elimination of natural selection for spawners with high lipid content, should be present in Fraser Chinook.

Beyond hatcheries, ocean conditions have been shown to impact fish, including salmon, lipid accumulation^46,59^. For example, Fraser sockeye have shown up to 15% lower energy levels in the runs following a year of low ocean productivity vs one of high productivity^49^. For migrating salmon themselves, particularly those from the Summer 4_1_ MU, which have a long migration during the summer season when river temperatures are greatest, and the Fall 4_1_ MU, which returns to the Fraser with the lowest mean lipid levels, such a decline could be significant for their own survival. For SRKWs, climate-driven decreases in average prey energy density could lead to energy stress and increased foraging effort. SRKWs forage on a diverse portfolio of Chinook with different marine life histories and distributions. Chinook diet is known to vary regionally and seasonally^60^, but how this variation interacts with lipid accumulation remains poorly understood. The impact of ocean conditions on the energy accumulation of these stocks will vary and needs to be considered in conservation planning for both species. Ongoing annual monitoring of Chinook MU-specific energy densities, aided by the large sample sizes possible with a microwave fat meter, is one means to observe and understand the effects of changing ocean conditions on Chinook energy densities.

## Conclusion

This study was initiated to evaluate Chinook salmon lipid content. We calibrated a method of rapid lipid assessment for Chinook salmon that is highly accurate and easily deployable and used it to derive estimates of lipid content and energy density for the five Fraser River Chinook MUs*.* Our data identified that early run Spring 5_2_ and Summer 5_2_ Chinook had the highest energy density of the Fraser River MUs, and that the Summer 4_1_ MU exhibited a unique and intermediate energy density prey source between the earlier runs and the least energy dense Fall 4_1_ MU. Changes to the abundance of Fraser River Chinook runs could significantly alter SRKW energy budgets. SRKWs typically rely on the Spring and Summer MUs for a majority of their summer diet, even as the energy density of arriving Chinook salmon drops over the season. In the late summer, fall and winter, SRKWs must rely on the lower energy Fall 4_1_ MU. Among Chinook stocks, variation in lipid content appeared to be driven by differences in migration distance and elevation. Within stocks, lipid content decreased across the migration season. This in-season lipid decrease was greater than would be expected from increased gonad maturation alone, suggesting a true effect of Chinook with greater lipid content returning to spawn earlier in the season than lower lipid content Chinook from the same population.

While the data collected in this study cannot resolve whether differences in stock-specific Chinook energy density are responsible for the decline/stagnation of SRKW populations, they do lay the groundwork to explore this in future work. Precise energy density estimates such as these can be used in tandem with known prey distribution data (e.g.^61^) and SRKW energetics models (e.g.^62^) to better understand how changes in MU population abundance and total size may affect the ability of SRKW to meet their energy requirements. These data can be used to prepare more targeted management strategies such as timed fisheries closures to protect specific MUs. Fisheries and Oceans Canada has implemented such a fisheries closure to protect the Summer 4_1_, Summer 5_2,_ and Spring 5_2_ MUs since 2019^63^ but this strategy does not extend to the United States, where these stocks are likely to be encountered as well. Other conservation schemes that these data inform include stock enhancement of more energy-rich Chinook populations, or the targeted rehabilitation of potentially more energy-rich wild populations in locations with high hatchery influence.

## Supplementary Information

Supplementary Information.

## Data Availability

The datasets used and analyzed during the current study are available in Zenodo at 10.5281/zenodo.7603999.
