# Supplementary material for: Seasonal variation in the lipid content of Fraser River Chinook Salmon (Oncorhynchus tshawytscha) and its implications for Southern Resident Killer Whale (Orcinus orca) prey quality
Source: Sci Rep. 2023 Feb 15;13:2675. doi: 10.1038/s41598-023-28321-9 (PMC9931693; doi:10.1038/s41598-023-28321-9)
Supplement: Supplementary file 1 — Supplementary Information. [file 41598_2023_28321_MOESM1_ESM.docx]

Supplementary

Table S1: Female/male gonad lipid content (% wet weight) and sample size (n).

| **Sex** | **Lipid Content (%)** | **N** |
| --- | --- | --- |
| Female | 10.0 ± 1.9 | 26 |
| Male | 0.68 ± 0.4 | 15 |


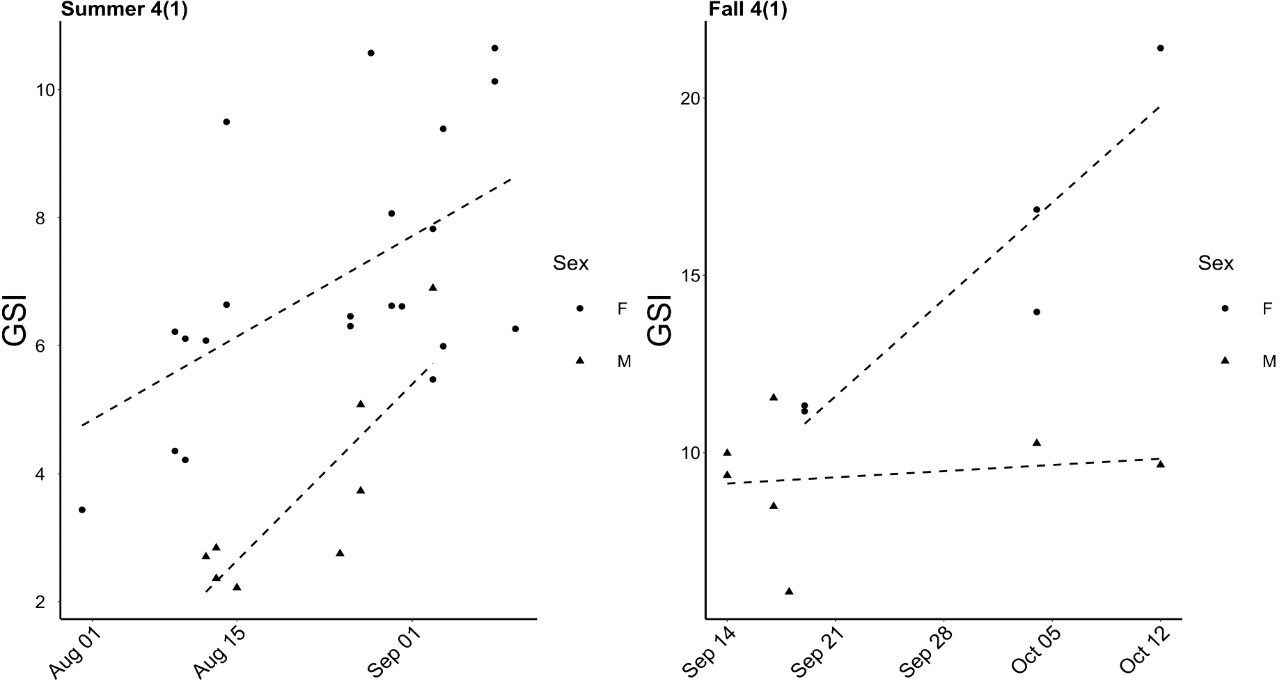


Figure S1: GSI over time for male and female Chinook from the Summer 4(1) and Fall 4(1) MUs.

Table S3: Adjusted R^2^ and slope of relationships between Fraser River Chinook Management Unit Lipid Content (% wet weight) and day of arrival, weight (kg), and Fork length (mm).

|  | Day of arrival | | Weight | | Fork length | |
| --- | --- | --- | --- | --- | --- | --- |
|  | R^2^ | slope | R^2^ | slope | R^2^ | slope |
| Spring 5_2_ | 0.4 | -0.07 | -0.03 | 0.03 | -0.002 | -0.03 |
| Summer 5_2_ | 0.2 | -0.07 | .007 | -0.09 | -0.01 | -.001 |
| Summer 4_1_ | 0.3 | -0.09 | .002 | .03 | -0.001 | -.00005 |
| Fall 4_1_ | 0.1 | -0.04 | 0.001 | -0.02 | -.00003 | -0.001 |


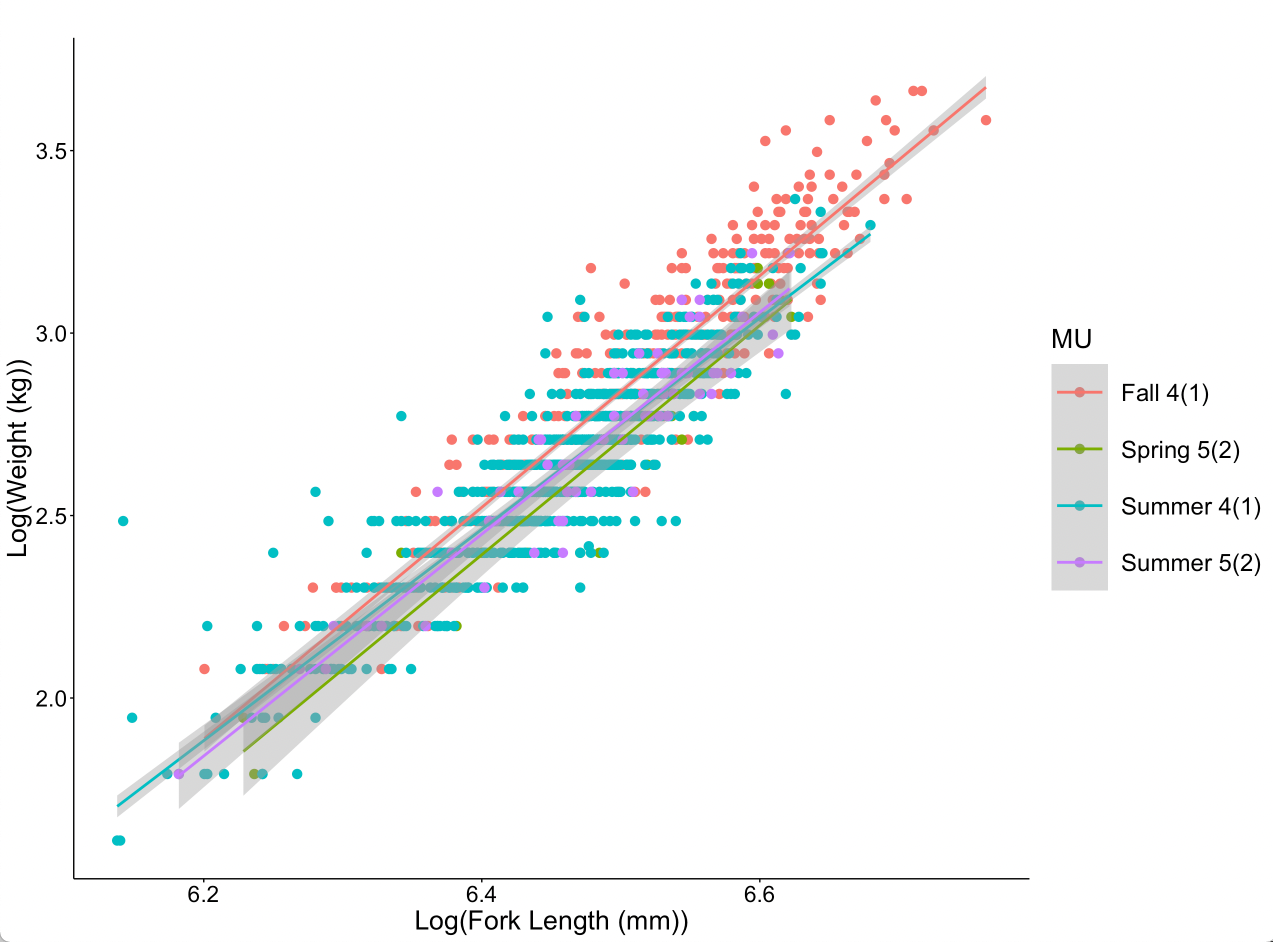


Figure S2: Plot of log(Fork Length (mm)) and Log(Weight (kg)) for four Fraser River Chinook MUs (Fall 4_1_, Summer 4_1_, Summer 5_2,_ and Spring 5_2_) fit with best-fit linear models.
